# Treatment of third-stage larvae of *Toxocara cati* with milbemycin oxime plus praziquantel tablets and emodepside plus praziquantel spot-on formulation in experimentally infected cats

**DOI:** 10.1007/s00436-012-3060-1

**Published:** 2012-08-04

**Authors:** Sonja Wolken, Claudia Böhm, Roland Schaper, Thomas Schnieder

**Affiliations:** 1 Institute for Parasitology, University of Veterinary Medicine Hannover, Foundation, Buenteweg 17, 30559 Hannover, Germany; 2 Bayer Animal Health GmbH, 51368 Leverkusen, Germany

## Abstract

*Toxocara cati* is the most prevalent gastrointestinal helminth in cats worldwide, with cats of all ages at risk of infection. An anthelminthic treatment that not only affects the gut-dwelling stages of this parasite but is also effective against developmental stages in the tissue has the advantage that the pathology caused by migrating larvae is minimized and the need for repeated treatments is reduced. This study was conducted to evaluate the efficacy of milbemycin oxime/praziquantel tablets (Milbemax®, Novartis) against third-stage larvae of *T. cati* in comparison to a spot-on formulation of emodepside and praziquantel (Profender®, Bayer). Twenty-four kittens were experimentally infected with *T. cati* and randomly allocated to three study groups. Treatments were performed at the minimum therapeutic dosage 5 days after the experimental infection. The development of patent infections was monitored and all cats were dewormed 50 days post-infection. Efficacies were calculated based on counts of excreted worms in the treated groups compared to a negative control group. Seven of the eight cats in the negative control group developed a patent *T. cati* infection and all cats were excreting worms at the end of the study (geometric mean worm count 18.1). No efficacy could be observed for the milbemycin oxime-treated animals. All cats developed a patent infection and excreted worms (geometric mean worm count 27.7). The treatment with Profender® was 98.5 % effective against L3 of *T. cati*. One cat developed a patent infection and was excreting worms at the end of the study (geometric mean worm count 0.3). No adverse reactions were noted in either treatment group.

## Introduction

Despite the availability of numerous highly efficacious anthelminthic products, worm infestations remain the most common finding in companion animals, with *Toxocara cati* being the most prevalent gastrointestinal helminth in cats worldwide. Analysis of lab data (Barutzki and Schaper 2003, 2011) in Germany showed that no significant changes in prevalence rates have been observed over the last 12 years. These results demonstrate the constantly high need for further efforts to control these pests and once again illustrate the parasites' effective survival strategies.

The high reproductive rate of ascarids and the extremely high tenacity of the eggs are major contributors to their success story. This indicates that strategic control of these parasites should minimize the contamination of the environment by animals that harbour patent infections. Besides this epidemiological aspect, it is also beneficial for the individual patient to be cleared of an existing infection as soon as possible to minimize the pathology caused by migrating larvae. In 1956, Sprent described the tracheal migration route of *T. cati* after egg ingestion and in this context observed the occurrence of haemorrhagic spots on the lungs. Later, it was shown that the pathological changes caused by migrating *T. cati* larvae include proliferation of the intima and media, which may result in complete occlusion of the pulmonary arteries (Swerczek et al. 1970; Weatherley and Hamilton 1984).

Since then, little attention has been paid to the pathology caused by early-migrating *T. cati* larvae, and currently, efficacy against third-stage larvae (L3) has been demonstrated only by one product (Profender®) (Reinemeyer et al. 2005). Profender® is thus the only product licenced for the treatment of these early stages of *T. cati*. Recently, the role that parasites plays in the development of acute and chronic lung disease in cats has been reinvestigated, and importance has been attached not only to the more obvious lung-relevant parasites like *Dirofilaria immitis* and *Aelurostrongylus abstrusus* but also to the migrating stages of *T. cati* (Dillon 2011).

This study aimed to evaluate and compare the efficacy of emodepside and the macrocyclic lactone milbemycin oxime against *T. cati* L3. For the latter, efficacy against gut-dwelling fourth-stage larvae has been demonstrated (Schenker et al. 2007), but no information is available on the efficacy against the stage of *T. cati* which affects the lungs.

## Materials and methods

The study was designed as a controlled, randomized and blinded efficacy study. Wherever possible, the principles of Good Clinical Practice (Veterinary International Conference on Harmonization (VICH) 2000a, b, c) and the WAAVP guideline for evaluating the efficacy of anthelmintics for dogs and cats (Jacobs et al. 1994) were followed.

Twenty-four purpose-bred domestic short-hair kittens (age 14–15 weeks) were acclimatized to the study facility for 7 days. During the acclimatization period, three faecal egg counts (FECs) were performed on consecutive days to demonstrate the absence of helminth infections. The kittens had never received an anthelminthic or other drug that could have interfered with the study objective. Fitness for study inclusion was demonstrated by physical examinations prior to the experimental infection and prior to treatment. On study day 0, all kittens were inoculated with approximately 400 embryonated *T. cati* eggs from a strain that had been maintained in the lab since 2010 and was originally isolated from a naturally infected cat in Germany.

Based on sex and body weight, the kittens were randomly allocated on study day 4 to three study groups with eight cats each and a sex ratio of 1:1 (Table 2). Treatments were performed on study day 5. Group 1 was treated with the minimum therapeutic dosage of 3 mg/kg emodepside plus 12 mg/kg praziquantel (Profender®), corresponding to 0.12 ml/kg spot-on solution. The commercially available 0.35-ml pipette was emptied into a glass vial and the appropriate volume was withdrawn using a 1,000-μl pipette and applied to the cat's skin at the base of the skull in front of the shoulder blades. Group 2 received the minimum therapeutic dosage of 2 mg/kg milbemycin oxime and 5 mg/kg praziquantel. The arithmetic mean weight of ten tablets of the batch was calculated to determine the correct tablet mass per kilogramme. Excess tablet mass was rubbed off with sandpaper. The maximum discrepancy between target and actual tablet weight was +0.8 mg/kg, corresponding to +0.02 mg/kg milbemycin oxime. Group 3 was the negative control group and was thus left untreated.

During the prepatent period, the kittens were group-housed in their respective treatment group. Housing and general maintenance was standardized across all animals. The cats received a commercially available dry food (Growth™, Royal Canin) and tap water ad libitum. General health observations were conducted daily during cleaning and feeding activities and during social interaction with the animal attendants. To monitor the possible onset of patency, FECs were performed on pooled group samples three times per week starting on study day 25. When the first pool of sample was positive, the cats were individually housed in cages and the number of eggs per gram of faeces was determined daily until the end of the study. Throughout the study, the rooms, cages and litter trays were carefully inspected to detect any spontaneously expelled worms. On study day 50, all cats were dewormed with a combination product of praziquantel and pyrantel (Drontal®) according to the manufacturer's recommendations, and all faeces were carefully searched for excreted worms for 3 days following the treatment. Worms were counted and differentiated by stage and sex and total numbers were used to calculate the product effect according to Abbot's formula for controlled tests:

$$ {\text{Percentage}}\;{\text{of efficacy}}\;(\% ) = 100 \times \left( {C - T} \right)/C $$

where *C* was the geometric mean of *T. cati* in the control group, and *T* was the geometric mean of *T. cati* in the treated group (Profender® or Milbemax®). To confirm the success of treatment, a final FEC was performed on study day 54 before the study was terminated. The experimental design is summarized in Table 1.

**Table 1:** Summary experimental design

Study day	Activity
−7 to −1	Acclimatization, FEC over 3 consecutive days
0	Experimental infection with 400 *T. cati* eggs
4	Randomization and allocation to groups
5	Treatment in groups 1 and 2
25—onset of positive group samples	Housing in groups, FEC three times a week in groups until positive, then individual housing
49—onset of positive group samples	FEC in individually housed cats
50	Anthelmintic treatment
51–53	Collection of worms in faeces
54	FEC

One-way analysis of variance was used to compare the groups with regard to pre-treatment body weights. A non-parametric test (Mann–Whitney rank sum test) was used to analyze worm count data and daily egg excretion.

## Results

All cats stayed clinically healthy throughout the study period. Treatment in groups 1 (Profender®) and 2 (Milbemax®) was tolerated well by all cats. Neither local nor systemic effects were observed. There was no statistically significant difference between the groups regarding body weight on study day 4 (*p* = 0.954).

**Fig. 1:**
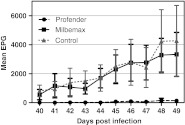
Arithmetic mean faecal egg count and standard deviation on the individual study days

On study day 36, the first positive faecal group sample was obtained in group 3 (negative control), and from study day 42 until the end of the study, seven out of eight cats in this group shed *Toxocara* eggs. One cat stayed coproscopically negative throughout the study. In group 2 (Milbemax®), all cats were coproscopically positive from study day 41 onwards. In group 1 (Profender®), seven out of eight cats stayed coproscopically negative throughout the study. One cat began to shed *Toxocara* eggs on study day 42. Where egg excretion was concerned, there was no statistically significant difference between group 2 (Milbemax®) and the control group (*p* ≥ 0.442). Egg counts in Profender®-treated animals differed significantly from the control group from study day 41 until the end of the study (*p* ≤ 0.035). After deworming at the end of the study, a total of 257 worms was collected in the negative control group. Worms were recovered from all eight cats, and six cats harboured an adequate infection (≥5 worms). The coproscopically negative cat harboured two male worms and an immature female. In the Milbemax®-treated group, a total of 318 worms was collected. Worm numbers in this treatment group exceeded the numbers in the control group; therefore, no treatment effect was evident. There was no statistically significant difference between the negative control group and the Milbemax®-treated group (*p* = 0.382). The treatment in group 1 (Profender®) was 98.5 % effective (*p* < 0.001). Only the coproscopically positive cat harboured six worms (Table 2). Spontaneously expelled worms were not detected in any group. Individual worm counts are presented in Table 3 and the course of egg excretion is presented in graph form in Fig. 1. The final FEC on study day 54 was negative in all cats, demonstrating the success of treatment.

**Table 2:** Group details

Group	Cat no.	Sex	Body weight on SD 4
1. Profender®	1	F	1.75
	2	F	1.65
	3	F	1.60
	4	F	1.20
	5	M	2.15
	6	M	2.00
	7	M	1.90
	8	M	1.45
2. Milbemax®	1	F	1.65
	2	F	1.60
	3	F	1.60
	4	F	1.30
	5	M	2.15
	6	M	2.10
	7	M	1.90
	8	M	1.35
3. Untreated control	1	F	1.80
	2	F	1.60
	3	F	1.55
	4	F	1.45
	5	M	2.30
	6	M	2.10
	7	M	1.70
	8	M	1.50

*SD* study day, *F* female, *M* male

**Table 3:** Individual worm counts

Group	Cat no.	No. of female *T. cati*	No. of male *T. cati*	No. of preadults	Total no. of *T. cati*	Geometric mean worm count
1. Profender®	1	0	0	0	0	0.3
	2	4	2	0	6	
	3	0	0	0	0	
	4	0	0	0	0	
	5	0	0	0	0	
	6	0	0	0	0	
	7	0	0	0	0	
	8	0	0	0	0	
2. Milbemax®	1	5	2	0	7	27.7
	2	6	5	0	11	
	3	10	22	10	42	
	4	9	30	7	46	
	5	9	13	0	22	
	6	55	53	27	135	
	7	11	7	2	20	
	8	17	18	0	35	
3. Untreated control	1	5	6	0	11	18.1
	2	7	18	0	25	
	3	1	2	0	3	
	4	1	3	0	4	
	5	8	6	4	18	
	6	50	54	8	112	
	7	14	27	24	65	
	8	5	14	0	19

## Discussion

The efficacy of the emodepside/praziquantel spot-on solution against third-stage larva of *T. cati* was comparable to the level of control obtained in the studies for marketing authorization (Reinemeyer et al. 2005). Efficacy calculations in the study presented here were based on worm counts after deworming and not on worm counts determined at necropsy. Although desirable from an ethical point of view, this procedure has disadvantages that reduce the reliability of the results and is hence generally not acceptable in laboratory studies for drug approval. Worms may be wholly or partially digested when passed in the faeces and may therefore not be identified. This fact is of lesser importance for “strong” worms like ascarids, but can be critical for worms that are more fragile. Further unintended loss of worms may occur through coprophagia, mainly seen in dogs, or ingestion of regurgitated worms as seen in dogs and cats. Finally, only a necropsy can prove that the animal was completely cleared of an existing worm burden. As seen in this study, low worm burdens or infections with single-sex worms or immature worms cannot be detected by faecal examinations. Despite these drawbacks, it can be concluded that reliable data were generated in this study. Results obtained in group 1 (Profender®) were similar to former studies and the negative control group showed an adequate level of infection. As is often seen in experimentally infected animals, the individual worm burdens in the negative control group were highly variable (range 3–112), but the requirement for six adequately infected animals was fulfilled.

No efficacy could be observed in the milbemycin oxime-treated cats. Numbers of excreted worms and eggs passed in the faeces exceeded those in the control group. Treatment was performed 5 days after the experimental infection to coincide with the migrating stage of the parasite (VICH 2000c). Although information on the detailed migratory behaviour of *T. cati* is scarce, observations by Sprent (1956) showed that after egg infection, the larvae perform a liver–lung migration and can be found in the lungs at this time point of infection. To be effective against these early-migrating larvae, an active ingredient has to be present at a sufficient level within these tissues and/or the blood stream at a certain time point. According to Reinemeyer and Courtney (2001), 5–10 % of an orally administered dose of milbemycin oxime is absorbed; the major part is excreted with the faeces. Although milbemycin oxime in the formulated tablet Milbemax® is effective against migrating larval stages of *D. immitis* in the cat (Genchi et al. 2004), the results obtained under the conditions of this study lead to the assumption that migrating *T. cati* larvae are not likewise affected, either due to low drug levels or to an unfavourable tissue distribution with regard to this parasite. It can be concluded that to date, the spot-on formulation of emodepside and praziquantel remains the only anthelmintic with proven efficacy against migrating *T. cati* larvae.
